# Evaluation of Adverse Effects of Mutein Forms of Recombinant Human Interferon Alpha-2b in Female Swiss Webster Mice

**DOI:** 10.1155/2013/943687

**Published:** 2013-05-02

**Authors:** H. Rachmawati, A. Merika, R. A. Ningrum, K. Anggadiredja, D. S. Retnoningrum

**Affiliations:** ^1^Research group of Pharmaceutics, School of Pharmacy, Bandung Institute of Technology, Ganesha 10, Bandung 40132, Indonesia; ^2^Research group of Pharmacology and Clinical Pharmacy, School of Pharmacy, Bandung Institute of Technology, Bandung 40132, Indonesia

## Abstract

*Purpose*. We successfully developed recombinant human interferon alpha-2b (rhIFN-**α**2b) and mutein forms through the site-directed mutagenesis technique. The mutein forms were developed by substituting cysteins at positions 2 and 99 with aspartic acids. The potential adverse effects of these rhIFN-**α**2bs were assessed by acute and subchronic studies. *Methods*. In the acute study, rhIFN-**α**2bs were subcutaneously administered to mice at a single dose of 97.5 **μ**g/kg, 975 **μ**g/kg, and 9.75 mg/kg BW and were observed for 14 days. In the subchronic study, single dose of 1.95 **μ**g/kg and 19.5 **μ**g/kg, respectively, was given subcutaneously every 3 days for 45 days. *Results*. No death as well as abnormality in body weight, behavior, presentation of main organs, and value of plasma SGPT and SGOT was observed. Wild type and mutein rhIFN-**α**2bs did not show significant adverse effects at dose up to 9.75 mg/kg BW. Administration of these rhIFN-**α**2bs given repeatedly did not induce any adverse effect. *Conclusion*. These results suggest that our rhIFN-**α**2bs are safe. However, further study is still needed to clarify the safety issue before use in clinical trial.

## 1. Introduction

Hepatitis B and C viruses are highly infectious, and the infections are transmitted almost 100 times more effective than HIV/AIDS. It attacks the liver and is a major cause of liver cancer. Of the estimated 50 million new cases of hepatitis B viral infection diagnosed annually, 5–10% of adults and up to 90% of infants will become chronically infected; 75% of these occur in Asia where hepatitis B is the leading cause of chronic hepatitis, cirrhosis, and hepatocellular carcinoma [1, 2]. Hepatitis C virus, referred to as parentally transmitted non-A non-B hepatitis, has been reported to be the most common transfusion-associated hepatitis in many countries worldwide. Hepatitis C virus infection has been characterized by longer incubation time and chronic, persistent infections and has sevenfolds increased risk of hepatocellular carcinoma [2–4]. Until recently, WHO recommends interferon alpha 2 as a standard therapy for hepatitis B/C virus, either as monotherapy or in combination with nucleotide/nucleoside analogs [5, 6].

Human alpha interferons (hIFN-*α*) comprise a family of closely related proteins that block viral infection, inhibit cell proliferation, and modulate cell differentiation. Recombinant hIFN-*α*2 has proven useful for the treatment of a variety of human viral diseases and cancers. However, the clinical use of this cytokine has been restricted due to its short circulating half-life, which makes frequent dosing over an extended period necessary. One main problem of using interferon as a therapy is that interferon exhibits short plasma half-life so that it is not effective to deliver sufficient amount to the target tissue. This short plasma half-life is commonly due to fast renal clearance which is related to the hydrophilic properties of this agent as well as its small size or to enzymatic degradation caused by enzymes present in blood, liver, and kidney. To improve its properties, several approaches have been performed such as chemical modification through pegylation and protein engineering [7]. Our report described a novel strategy on this protein by replacing nonpotential aminoacids influencing the biological activity of the protein with more negative aminoacid, hence increasing the net negative charge of the protein.

Protein charge is a crucial factor for kidney elimination, hence; any effort of modifying this charge will alter its half-life. Negatively charged proteins will be repulsed by glomerular cells that are positively charged. Therefore, the negatively charged proteins will be recirculated into the blood. Diminished kidney elimination and increased blood circulation will prolong protein half-life. This in turn will decrease protein dosing and consequently will lower its toxicity [8].

There are 12 aminoacids (Leu30, Lys31, Arg33, His34, Phe36, Arg120, Lys121, Gln124, Tyr122, Tyr129, Lys131, and Glu132) in hIFN-*α*2b that are involved in its biological activity [9]. Among 4 cysteine residues, cysteines at positions 1 and 98 which form a disulfide bridge are not required for biological activity of IFN-*α*2b [10]. Even the disruption of this disulfide bridge by serine substitutions resulted in higher antiviral activity [11]. We have previously cloned the gene encoding wild type human IFN-*α*2b using synthetic gene approach and have successfully overproduced the protein in *Escherichia coli * [12, 13]. The recombinant protein has been purified and confirmed by nano-LC mass spectrometry to be the right IFN-*α*2b. We further engineered the protein to generate rhIFN-*α*2bs that are more negatively charged than the wild type counterpart. The technique developed was site-directed mutagenesis of gene encoding IFN-*α*2b. The site-directed mutagenesis was focused on two cystein codons (at positions 2 and 99) to be replaced by aspartic acids using mutagenic primers. Our previous results demonstrated that all engineered rhIFN-*α*2bs (C_2_D, C_99_D, C_2_DC_99_D) exhibited longer elimination half-life than their wild type counterpart [14]. The aim of this study was to evaluate the safety of our novel IFN-*α*2bs. As an earlier step on this matter, we studied whether any potential adverse effects developed on these mutein forms when administered in higher dose for longer period of time.

## 2. Materials and Methods

### 2.1. Bacterial Strains and Culture Media

*E. coli *strain BL21(DE3) containing IFN-*α*2b wild type and muteins open reading frame previously constructed was used for gene expression [12]. Luria-Bertani (LB) broth and agar containing 100 *μ*g/mL of ampicillin was applied for bacterial growth, and 0.5 mM of isopropyl d-1-thiogalactopyranoside (IPTG) was for protein overproduction.

### 2.2. Interferon Preparation

The wild type and a mutein IFN-*α*2b (C_2_DC_99_D) were each overproduced using optimized condition previously described [12, 13]. The crude and purified proteins (as soluble protein) were analyzed using 15% sodium dodecyl sulphate polyacrylamide gel electrophoresis (SDS-PAGE) under denaturing condition. All IFN-*α*2bs were affinity purified using Nickel column, and imidazole was removed from purified protein using nanosep centrifugal concentration with 10 kDa cutoff (Pall Life Science) [12]. Protein concentration was determined using Bradford method based on coomassie blue staining. 

### 2.3. Animals

Female Swiss Webster mice, 2-3 months of age, were obtained from the Animal Laboratory, School of Pharmacy, Bandung Institute of Technology, Indonesia. The mice were housed under the barrier-sustained condition in a well-ventilated animal room according to standard laboratory condition with free access to standard diet. Animals used in the experiments received care in compliance with the “Principles of Laboratory Animal Care” and “Guide for the Care and Use of Laboratory Animals.” They were acclimatized for about 1 week prior to administration of the test substances. Standard mouse pellet and tap sterilized water were given ad libitum. 

### 2.4. Experimental Design

Studies were conducted under Good Laboratory Practice conditions at Animal Laboratory School of Pharmacy, Bandung Institute of Technology, Bandung, Indonesia. Animals were sacrificed under anaesthesia with 40% O_2_ : 60% N_2_O combined with 0.5% Isoflurane (Abbot Laboratoriesb Ltd. Queensborough, Kent, UK).

### 2.5. Evaluation of Acute Adverse Effect

Acute and subchronic adverse effects study was performed according to OECD Guide Lines for the Testing of Chemicals, Test no. 425 (2008). Thirty-five female mice were divided into 3 groups: control and 2 test groups each receiving either rhIFN-*α*2b wild type or mutein. The mice in test groups were further divided into 3 groups, each was given 97.5 *μ*g/kg, 975 *μ*g/kg, or 9.75 mg/kg BW rhIFN-*α*2b in a single subcutaneous injection. The control group only received similar treatment with water for injection. Observations of pharmacotoxic signs were carried out at 0.5, 1, 2, 4, and 24 h after dosing during the first day and daily thereafter for 14 days. The time of onset, intensity, and duration of these symptoms, if any, was recorded. All animals were observed twice daily for mortality during the 14-day period of study. The weight of each mouse was recorded daily throughout the course of the study. The group's mean body weights were calculated. At the termination of the study (14 days), all animals were sacrificed, and a complete organ analysis with both macroscopic and microscopic observations was performed. Histological analysis was carried out using haematoxylin/eosin staining on the vital organs including liver, kidney, and heart. 

### 2.6. Evaluation of Subchronic Adverse Effect

Fifty-four female mice were divided into five groups: control; two test groups, each receiving either rhIFN-*α*2b wild type or mutein, and two satellite groups. Mice in test groups were further divided into two groups receiving either 1.95 *μ*g/kg or 19.5 *μ*g/kg BW of interferons. The interferons were given subcutaneously every 3 days as a single dose during a 45-day period. The control group only received water for injection through the same route.

At the end of the 45-day period, the animals were fasted overnight. In the following morning, blood sample was collected from the retro-orbital sinus. All animals except the satellite groups were then sacrificed. Vital organs including liver, kidney, lymph, ovary, uterus, and heart were isolated and analyzed. Both macroscopic and microscopic observations were performed in a similar way to those performed in acute study. In addition, the activities of serum glutamic pyruvic transaminase (SGPT) and serum glutamic oxaloacetic transaminase (SGOT) were assayed. Animals in satellite groups were allowed to survive to check the reversibility of the adverse effects. 

### 2.7. Statistical Analysis

The data which were expressed as mean ± standard deviation were analyzed using analysis of variance (ANOVA). Significant difference was considered if *P* < 0.05.

## 3. Results

### 3.1. SDS-PAGE Analysis of Wild Type and Mutein Forms of Interferon Alpha-2bs

**Figure 1:**
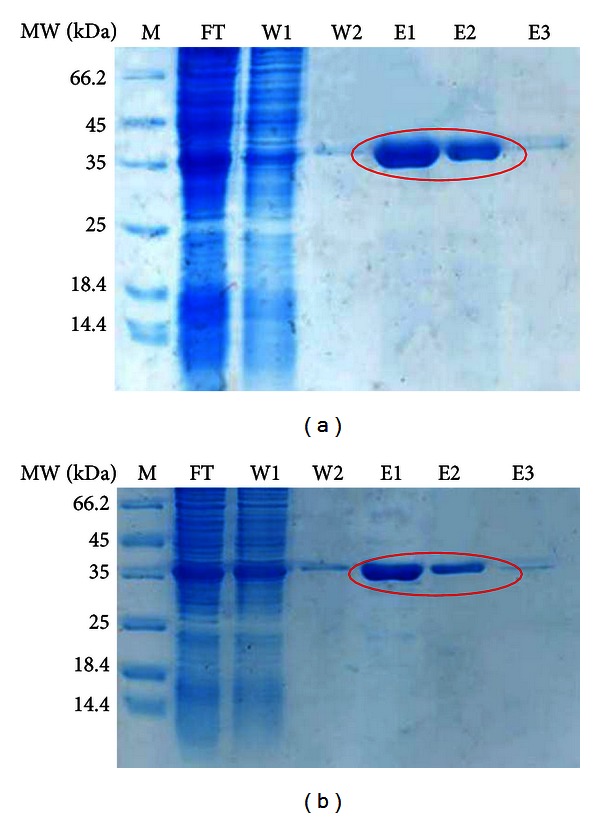
SDS-PAGE analysis of wild type rIFN-*α*2b and rIFN-*α*2bC_2_DC_99_D. (a) Wild type rIFN-*α*2b; (b) rhIFN-*α*2bC_2_DC_99_D; M: protein marker; FT: flow through; W1: washing 1; W2: washing 2; E1: elution 1; E2: elution 2; E3: elution 3.

Figure 1 shows a single band of wild type and mutein forms of rhIFN-*α*2b; all exhibited correct size as calculated. SDS-PAGE analysis was performed to check the quality of proteins prior to the study.

### 3.2. Acute Adverse Effect Evaluation

The aim of this evaluation was to observe the potential adverse effect on the wild type as well as the mutein form of interferon alpha-2b during short period of administration. 

#### 3.2.1. Mortalities

There was no death observed in all groups (Table 1). Therefore, the LD_50_ value in mice was higher than 9.75 mg/kg BW which is equivalent to 2500 times therapeutic dose of interferon through subcutaneous administration.

#### 3.2.2. Behavioral Findings

The parameters observed during the study included performances in general behavior, respiratory, autonomic, and central nervous systems, digestive system (defecation and urination), and the motor activity. No unusual changes were observed in behavior or locomotor activity. Furthermore, no ataxia and signs of intoxication were found in all groups during the 14-day observation period. 

#### 3.2.3. Body Weight

**Figure 2:**
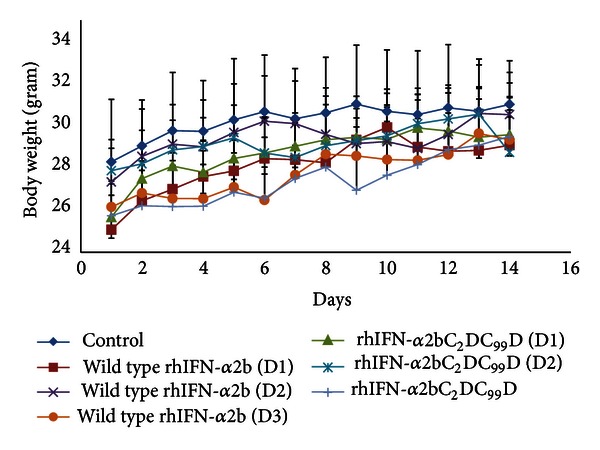
Body weight of the mice after a single dose administration of wild type rIFN-*α*2b and mutein rIFN-*α*2bC_2_DC_99_D.

Decrease in body weight can be an early sign of toxic effect of the test drugs. In this study, no significant difference was observed in the body weights between treated and the control groups (Figure 2). 

#### 3.2.4. Organ Indices

Organ indices refer to the weight of the organ(s) relative to the body weight. This observation was performed at the end of the study to check whether any alteration occurred on vital organs after the animal received the rhIFN-*α*2bs. As shown in Table 2, in particular for liver and kidney, there was an increase in organ index when the mice were given the highest dose of both forms of interferon. However, this difference was not significant statistically (*P* > 0.05).

#### 3.2.5. Microscopic Observation

**Figure 3:**
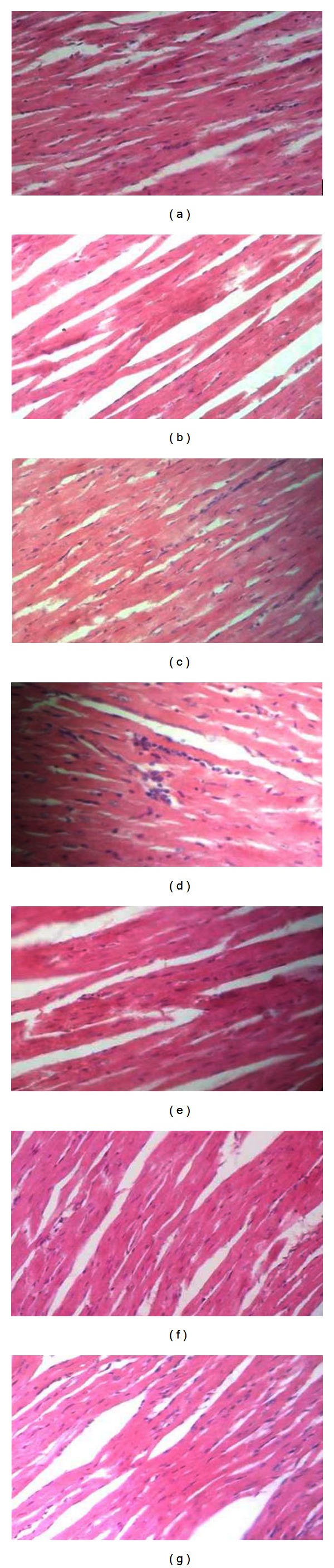
Histological presentation after H/E staining of heart tissue of the mice treated with a single dose of subcutaneous wild type rIFN-*α*2b or rhIFN-*α*2bC_2_DC_99_D (magnification 400x). (a) Control; (b) wild type rhIFN-*α*2b (D1); (c) rhIFN-*α*2bC_2_DC_99_D (D1); (d) wild type rhIFN-*α*2b (D2); (e) rhIFN-*α*2bC_2_DC_99_D (D2); (f) wild type rhIFN-*α*2b (D3); (g) rhIFN-*α*2bC_2_DC_99_D (D3).

**Figure 4:**
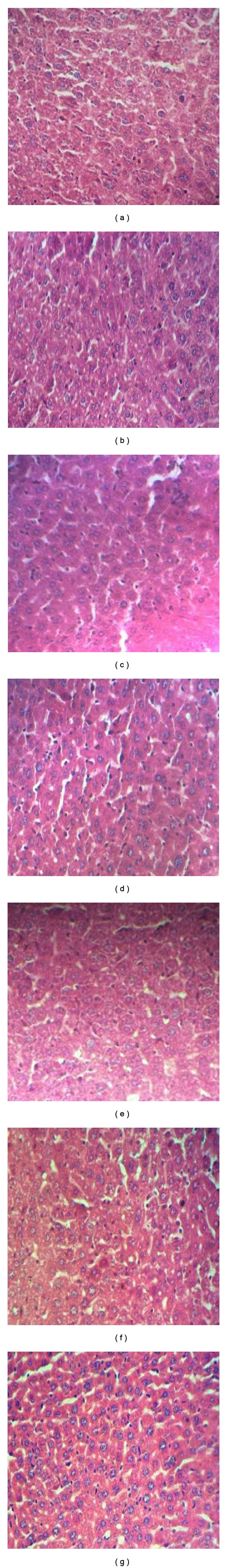
Histological presentation after H/E staining of liver of the mice treated with a single dose of subcutaneous wild type rhIFN-*α*2b or rhIFN-*α*2bC_2_DC_99_D (magnification 400x). (a) Control; (b) wild type rhIFN-*α*2b (D1); (c) rhIFN-*α*2bC_2_DC_99_D (D1); (d) wild type rhIFN-*α*2b (D2); (e) rhIFN-*α*2bC_2_DC_99_D (D2); (f) wild type rhIFN-*α*2b (D3); (g) rhIFN-*α*2bC_2_DC_99_D (D3).

**Figure 5:**
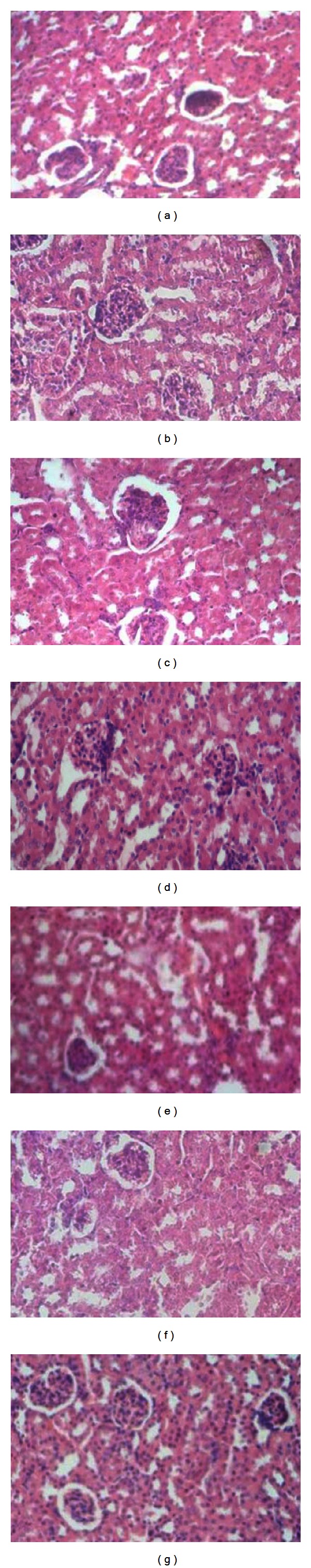
Histological presentation after H/E staining of kidney of the mice treated with a single dose of subcutaneous wild type rhIFN-*α*2b or rhIFN-*α*2bC_2_DC_99_D (magnification 400x). (a) Control; (b) wild type rhIFN-*α*2b (D1); (c) rhIFN-*α*2bC_2_DC_99_D (D1); (d) wild type rhIFN-*α*2b (D2); (e) rhIFN-*α*2bC_2_DC_99_D (D2); (f) wild type rhIFN-*α*2b (D3); (g) rhIFN-*α*2bC_2_DC_99_D (D3).

As shown in Figures 3, 4, and 5, there were no significant differences in the microscopic evaluation of the heart, liver, and kidney between control and treated groups.

### 3.3. Subchronic Adverse Effect Evaluation

#### 3.3.1. Behavioral Findings

The parameters observed during the study included performances in general behavior and body weight. As previously recorded in acute study, no unusual changes in behavior or locomotor activity were found, and no signs of intoxication were seen in all groups during the 45-days observation period. 

#### 3.3.2. Body Weights and Organ Indices

**Figure 6:**
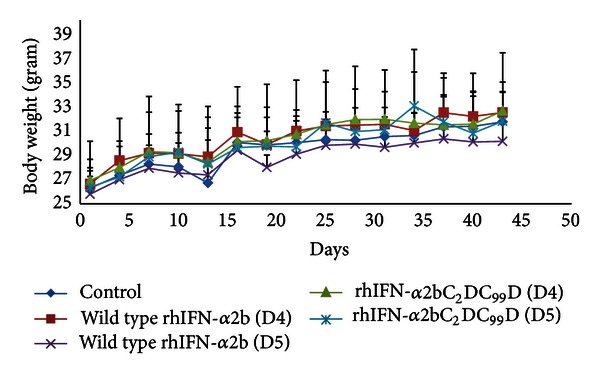
Body weight of the mice after subcutaneous administration of wild type rhIFN-*α*2b and rhIFN-*α*2bC_2_DC_99_D once every 3 days in the subchronic study period that lasted for 45 days.

As observed in the acute adverse effect evaluation, no differences were found in body weight as well as organ indices between mice in the control group and those treated with various doses of wild type or mutein forms of rhIFN-*α*2bs (Figure 6 and Table 3). 

#### 3.3.3. Microscopic Observation

**Figure 7:**
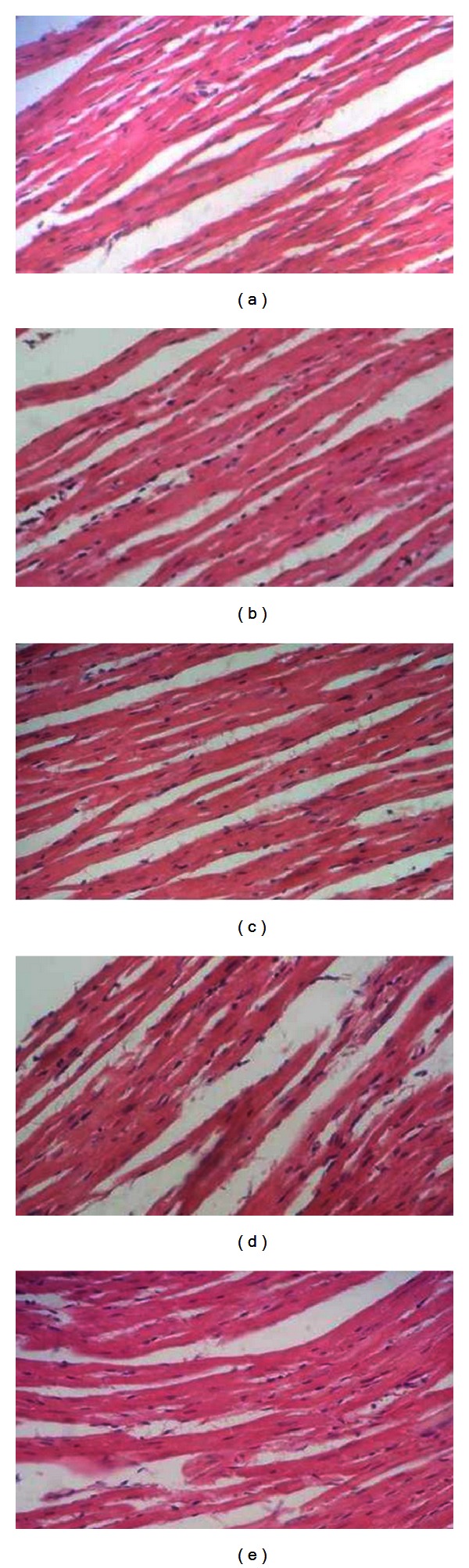
Histological presentation of H/E staining of heart tissue of the mice after subcutaneous administration of wild type rhIFN-*α*2b or rhIFN-*α*2bC_2_DC_99_D once every 3 days during a 45-day period (magnification 400x). (a) Control; (b) wild type rhIFN-*α*2b (D4); (c) rhIFN-*α*2bC_2_DC_99_D (D4); (d) wild type rhIFN-*α*2b (D5); (e) rhIFN-*α*2bC_2_DC_99_D (D5).

**Figure 8:**
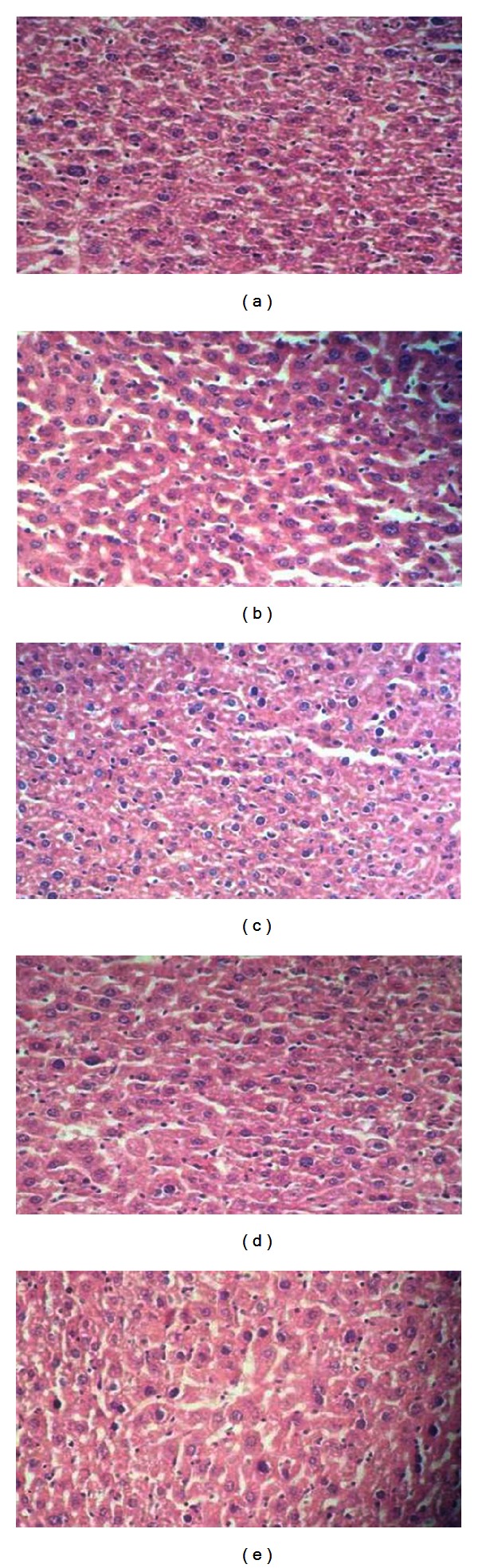
Histological presentation of H/E staining of liver tissue of the mice after subcutaneous administration of wild type rhIFN-*α*2b or rhIFN-*α*2bC_2_DC_99_D once every 3 days during a 45-day period (magnification 400x). (a) Control; (b) wild type rhIFN-*α*2b (D4); (c) rhIFN-*α*2bC_2_DC_99_D (D4); (d) wild type rhIFN-*α*2b (D5); (e) rhIFN-*α*2bC_2_DC_99_D (D5).

**Figure 9:**
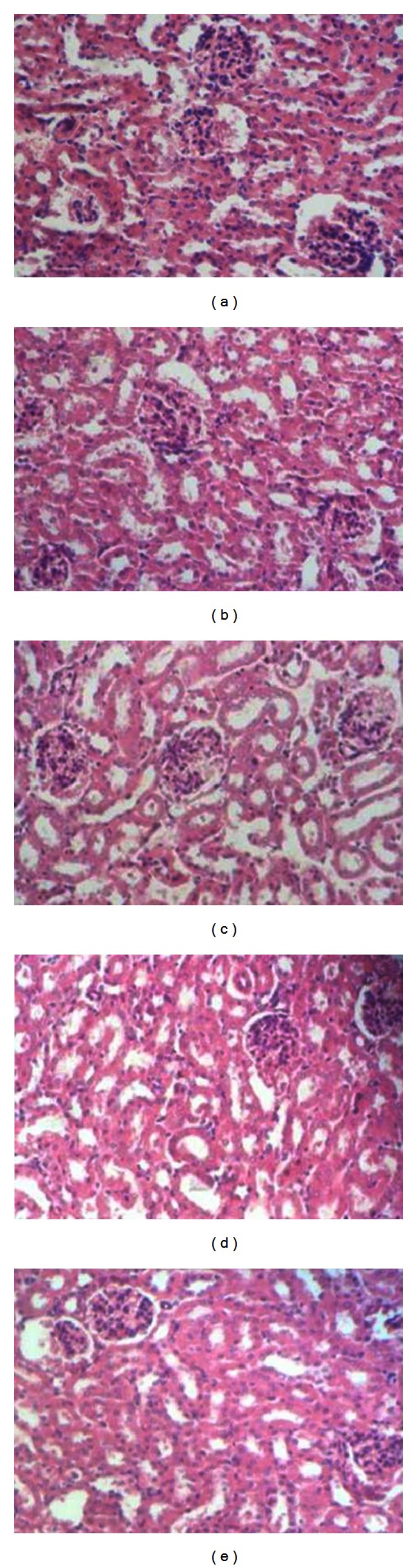
Histological presentation of H/E staining of kidney tissue of the mice after subcutaneous administration of wild type rhIFN-*α*2b or rhIFN-*α*2bC_2_DC_99_D once every 3 days during a 45-day period (magnification 400x). (a) Control; (b) wild type rhIFN-*α*2b (D4); (c) rhIFN-*α*2bC_2_DC_99_D (D4); (d) wild type rhIFN-*α*2b (D5); (e) rhIFN-*α*2bC_2_DC_99_D (D5).

H/E staining analysis as seen in Figures 7, 8, and 9 clearly shows no difference in the histology of heart, liver, and the kidney tissues between control and treated groups. This was in line with the observations of gross pathology performed in all mice immediately after dissection, demonstrating the uniformity in health, and the lack of any apparent pathological abnormalities. These results indicated that administration of these rhIFN-*α*2bs at the tested doses did not result in any adverse toxicological effects on these organs.

Table 4 shows the activities of two major hepatic enzymes analyzed in plasma. No significant differences were observed in the enzyme activities between the control and rhIFN-*α*2bs-treated groups. SGPT and SGOT are the intracellular enzymes expressed constitutively with biochemical catalytic functions in the cells. Induction of these enzymes may indicate the imbalance in the liver function due to pathological stress.

## 4. Discussion

The purpose of this study was to assess the safety of our rhIFN-*α*2bs either as wild type or mutein form when they were given acutely or repeatedly. The rhIFN-*α*2bs expressed from synthetic gene and in particular the novel mutein form (rhIFN-*α*2bC_2_DC_99_D) used in this study is of chemically unique. Unlike other engineered rhIFN-*α*2bs, C_2_DC_99_D has only one disulphide bridge due to aspartic acid substitutions on cysteines 2 and 99 and therefore has more negative charge. A distinguishing feature of this mutein form of rhIFN-*α*2b is the lack of charged-mediated renal clearance, which is expected to prolong its presence in the systemic circulation. This charge-modified type is expected to have improved pharmacokinetic profile compared to the wild type form of the interferon [14].

The acute adverse effect evaluation revealed that the value of LD_50_ was higher than 9.75 mg/kg BW for the tested rhIFN-*α*2b which is equivalent to 2500 times as much therapeutic human dose of interferon given subcutaneously. This result further corroborates a previous study (personal communication) showing no acute toxicological reaction or death when interferon was administered i.v. or intraabdominally to mice at doses up to 10^4^ to 10^5^ times of human dosage. Furthermore, recent work showed no effect on general signs, body weight, food consumption, and blood chemistry when interferon was given acutely to rat and monkey [11].

Our present results further showed that repeated administration of the rhIFN-*α*2bs did not induce significant changes in behavioral findings, blood chemistry, and histological presentation of the main organs. Again, these results further supported previous works demonstrating the good tolerability of interferon. Thus, Meng et al. [15] showed that after repeated dose of IFNalpha-2a-NGR (Asn-Gly-Arg), all the clinical chemistry changes were of minor severity there were no pertinent abnormal parameters or results observed [15]. Furthermore, these authors found that the increase in spleen and thymus organ-to-body weight ratios and decrease in menses were mild, reversible, and likely related to the pharmacology of the interferon. In addition, a recent study showed that administration of up to 100 *μ*g/kg (11 MIU/kg) PEG (Polyethylene Glycol)-IFN *β*-1a given subcutaneously or intramuscularly once weekly for 5 weeks in monkeys resulted in no drug-related adverse effects.

As indicated previously, the good safety feature of our modified interferons might be closely related to the improved pharmacokinetic properties, affecting mainly the distribution and elimination of the substance. Indeed, Hu and colleagues [16] have reported that polyethylene glycol- (PEG-) IFN *β*-1a showed greater exposure, longer half-life, lower clearance, and reduced volume of distribution than unmodified IFN *β*-1a, and this was accompanied with the elevation of neopterin (pharmacodynamic marker of interferon) concentration [16]. In line with this finding, it has been demonstrated that anchoring IFN-*α* to ApoA-I prolongs the half-life of IFN-*α* and promotes targeting to the liver, which might be responsible for the increased immunostimulatory properties and lower hematological toxicity [17].

In conclusion, results of our present work demonstrated that the wild type interferon as well as the novel mutein that we developed does not result in adverse effects and is well tolerated when administered during a 45-day study period, which suggests the potential for their safe use. Further work toward evaluating their efficacy is in progress and shall be reported elsewhere.

## Figures and Tables

**Table 1:** Mortalities and LD_50_ values of female Swiss Webster mice after a single subcutaneous administration of wild type rIFN-*α*2b or mutein rIFN-*α*2bC_2_DC_99_D.

Group	Substances	*N*	Σ mortality	Response (%)	LD_50_
Control	Water for injection	6	0	0	>9.75 mg/kg BW
I	Wild type rIFN-*α*2b D1	6	0	0	
II	Wild type rIFN-*α*2b D2	6	0	0	
III	Wild type rIFN-*α*2b D3	6	0	0	
IV	rIFN-*α*2bC_2_DC_99_D D1	6	0	0	
V	rIFN-*α*2bC_2_DC_99_D D2	6	0	0	
VI	rIFN-*α*2bC_2_DC_99_D D3	6	0	0	

D1 = 97.5 *μ*g/kg BW, D2 = 975 *μ*g/kg BW, and D3 = 9.75 mg/g BW.

**Table 2:** Organ indices of female mice after a single dose of wild type rIFN-*α*2b or rIFN-*α*2bC_2_DC_99_D.

Organ	Control	Groups					
		Wild type rIFN-*α*2b (D1)	rIFN-*α*2bC_2_DC_99_D (D1)	Wild type rIFN-*α*2b (D2)	rIFN-*α*2bC_2_DC_99_D (D2)	Wild type rIFN-*α*2b (D3)	rIFN-*α*2bC_2_DC_99_D (D3)
Heart	0.43 ± 0.04	0.45 ± 0.03	0.42 ± 0.02	0.43 ± 0.04	0.44 ± 0.04	0.43 ± 0.04	0.42 ± 0.04
Liver	4.91 ± 0.97	4.18 ± 1.81	4.57 ± 0.27	4.94 ± 0.50	5.53 ± 0.30	5.27 ± 0.50	5.05 ± 0.91
Kidney	1.07 ± 0.14	1.39 ± 0.09	1.17 ± 0.04	1.15 ± 0.11	1.12 ± 0.09	1.25 ± 0.15	1.16 ± 0.08
Limp	0.37 ± 0.14	0.36 ± 0.05	0.33 ± 0.06	0.34 ± 0.04	0.42 ± 0.13	0.63 ± 0.14	0.65 ± 0.28
Ovary-uterus	0.62 ± 0.21	0.73 ± 0.17	0.65 ± 0.11	0.63 ± 0.21	0.62 ± 0.15	0.62 ± 0.08	0.80 ± 0.26
Lung	0.54 ± 0.06	0.62 ± 0.09	0.54 ± 0.04	0.57 ± 0.12	0.58 ± 0.12	0.62 ± 0.06	0.59 ± 0.07

**Table 3:** Organ indices of female mice after subcutaneous administration of wild type rIFN-*α*2b or rIFN-*α*2bC_2_DC_99_D once every 3 days during a 45-day period.

Organ	Control	Groups			
		Wild type rIFN-*α*2b (D4)	rIFN-*α*2bC_2_DC_99_D (D4)	Wild type rIFN-*α*2b (D5)	rIFN-*α*2bC_2_DC_99_D (D5)
Heart	0.43 ± 0.03	0.41 ± 0.013	0.41 ± 0.04	0.43 ± 0.03	0.41 ± 0.03
Liver	5.81 ± 0.39	5.95 ± 0.33	6.14 ± 1.06	5.97 ± 0.55	5.39 ± 0.26
Kidney	1.37 ± 0.16	1.39 ± 0.16	1.48 ± 0.07	1.34 ± 0.05	1.30 ± 0.12
Limp	0.66 ± 0.15	0.59 ± 0.14	0.72 ± 0.18	0.58 ± 0.14	0.48 ± 0.09
Ovary-uterus	0.69 ± 0.16	0.64 ± 0.12	0.78 ± 0.14	0.72 ± 0.19	0.69 ± 0.19
Lung	0.59 ± 0.07	0.58 ± 0.02	0.60 ± 0.09	0.61 ± 0.05	0.59 ± 0.07

D4 = 1.95 *μ*g/kg BW, D5 = 19.5 *μ*g/kg BW.

**Table 4:** Enzyme activities in plasma of the mice after injection of the interferons once every 3 days during a 45-day period.

	Control	Wild type rIFN-*α*2b (D4)	rIFN-*α*2bC_2_DC_99_D (D4)	Wild type rIFN-*α*2b (D5)	rIFN-*α*2bC_2_DC_99_D (D5)
SGPT	54 ± 2	50.67 ± 14.04	54 ± 1	50.33 ± 16.01	53.33 ± 22.36
SGOT	215.67 ± 24.58	231 ± 19.52	219.67 ± 44.76	215.67 ± 41.88	205 ± 44.03
